# Effectiveness of Technology Interventions in Addressing Social Isolation, Connectedness, and Loneliness in Older Adults: Systematic Umbrella Review

**DOI:** 10.2196/40125

**Published:** 2022-10-24

**Authors:** Eric Balki, Niall Hayes, Carol Holland

**Affiliations:** 1 Centre for Ageing Research Faculty of Health and Medicine Lancaster University Lancaster United Kingdom; 2 Nottingham Trent University Nottingham United Kingdom

**Keywords:** information and communications technology, interventions, loneliness, older adults, social connectedness, social isolation, technology interventions

## Abstract

**Background:**

The global population of older adults (aged >60 years) is expected to triple to 2 billion by 2050. Proportionate rises in older adults affected by loneliness and social isolation (or social connectedness) are expected. Rapid deployability and social changes have increased the availability of technological devices, creating new opportunities for older adults.

**Objective:**

This study aimed to identify, synthesize, and critically appraise the effectiveness of technology interventions improving social connectedness in older adults by assessing the quality of reviews, common observations, and derivable themes.

**Methods:**

Following the guidelines of PRISMA (Preferred Reporting Items for Systematic Reviews and Meta-Analyses), 4 databases (PsycINFO, PubMed, Embase, and MEDLINE) were searched between February 2020 and March 2022. We identified reviews with adults aged ≥50 years in community and residential settings, reporting outcomes related to the impact of technologies on social disconnectedness with inclusion criteria based on the population, intervention, context, outcomes, and study schema—review-type articles (systematic, meta-analyses, integrative, and scoping)—and with digital interventions included. Grading of Recommendations, Assessment, Development, and Evaluations (GRADE) was used to measure the strength of outcome recommendations including the risk of bias. The reviews covered 326 primary studies with 79,538 participants. Findings were extracted, synthesized, and organized according to emerging themes.

**Results:**

Overall, 972 publications met the initial search criteria, and 24 met our inclusion criteria. Revised Assessment of Multiple Systematic Reviews was used to assess the quality of the analysis. Eligible reviews (3/24, 12%) were excluded because of their low Revised Assessment of Multiple Systematic Reviews scores (<22). The included reviews were dedicated to information and communications technology (ICT; 11/24, 46%), videoconferencing (4/24, 17%), computer or internet training (3/24, 12%), telecare (2/24, 8%), social networking sites (2/24, 8%), and robotics (2/27, 8%). Although technology was found to improve social connectedness, its effectiveness depended on study design and is improved by shorter durations, longer training times, and the facilitation of existing relationships. ICT and videoconferencing showed the best results, followed by computer training. Social networking sites achieved mixed results. Robotics and augmented reality showed promising results but lacked sufficient data for informed conclusions. The overall quality of the studies based on GRADE was medium low to very low.

**Conclusions:**

Technology interventions can improve social connectedness in older adults. The specific effectiveness rates favor ICT and videoconferencing, but with limited evidence, as indicated by low GRADE ratings. Future intervention and study design guidelines should carefully assess the methodological quality of studies and the overall certainty of specific outcome measures. The lack of randomized controlled trials in underlying primary studies (<28%) and suboptimal methodologies limited our findings. Robotics and augmented or virtual reality warrant further research. Low GRADE scores highlight the need for high-quality research in these areas.

**Trial Registration:**

PROSPERO CRD42022363475; https://tinyurl.com/mdd6zds

## Introduction

### Background

The use of technology to support older adults against feelings of loneliness and social isolation provides novel opportunities that have grown in the field of aging, as technology demonstrates that information and communications technology (ICT) use and training [1] and robotics conflate in the provision of programs and activities to facilitate social connectedness.

Social isolation and loneliness in older adults have been extensively researched. Many studies showed that the prevalence of these problems increases with age. For example, the prevalence of loneliness among young adults, early to middle–aged adults, and late to middle–aged older adults are 39.7%, 43.3%, and 48.2%, respectively [2]. The current global population of people aged ≥60 years is expected to triple to 2 billion by 2050 [3]. The number of people aged >50 years experiencing loneliness is expected to reach 2 million by 2025-2026, a 49% increase in 10 years [1]. Loneliness and social isolation are different concepts but are interlinked and can be considered the constructs of social disconnectedness [4]. Social isolation is objectively defined as the deprivation of relationships and social interactions, whereas loneliness is a subjective sense of not meeting one’s social needs [5]. Socially disconnected individuals are vulnerable to social isolation and loneliness because they have small social networks and low participation rates in social activities [6]. Fafchamps and Shilpi [7] defined social isolation as “deprivation of social connectedness and an inadequate quality and quantity of social relations at different levels of interactions (individual, group, community and broader social environment)” [6].

Socially disconnected older adults are also vulnerable to a range of health disorders, including infection [8], high blood pressure [9], impaired cognitive function [10], depression [11], stress associated elevation of hypothalamic-pituitary-adrenocortical  activity  [12], cardiovascular disease [13], diminished immunity [14], and mortality [15]. In addition, loneliness elevates the risk of dementia [16] and accelerates the progression of Alzheimer disease [10]. As the population proportion of older adults increases, negative health outcomes are expected to rise along with social isolation, and loneliness is likely to increase along with negative health outcomes [17].

Rapidly deployable technologies, along with socioeconomic changes that have reduced the cost of technology, have increased the accessibility of technological devices, creating new opportunities for older adults [18]. Internet-based technology interventions for social disconnectedness have grown over the past decade [19]. Digital communication technologies can improve the lives of older adults by facilitating their social relationships. Technologies such as email, social networking sites (SNSs), videoconferencing, and mobile instant messaging (MIM) apps have been shown to improve self-rated health and lower the incidence of loneliness, chronic illnesses, and depressive symptoms in older adults [20]. They also supplement the social benefits of physical interactions by reinforcing existing connections or providing routes to new connections, further reducing loneliness levels. Frequent users of technology and the internet can also access health information and social support for psychosocial problems. However, many studies on technology intervention ignore confounding factors, such as age, gender, living arrangements, economic status, education level, cognitive status, and daily living activities [21,22], which may influence the effectiveness of the intervention and the robustness of the findings. The small number of high-quality studies in this arena limits the generalizability of the results.

Several reviews have summarized works on technology interventions for older adults experiencing loneliness [23,24], but their value is diminished by the plethora of unclear evidence, heterogeneity of both populations, measures and methodologies, diverse outcomes, scattered focus, and broad topics. As the existing reviews are heterogeneous in content, lacking the investigation of outcome measures used and discussions on causation, they cannot reach generalizable conclusions.

For a standardized systematic report on these reviews, we must assess the quality of the reviews and find common observations and derivable themes. An umbrella review method can provide a focus for areas where there are competing interventions and amalgamate evidence from multiple quantitative and qualitative reviews [25]. To our knowledge, an umbrella review exploring the types and effectiveness of intervention technologies for social connectedness has not been published.

### Aims

To bridge this gap in the literature, we aimed to explore the findings and limits of current knowledge on the impact of technology interventions on social disconnectedness in older adults. We also emphasize areas requiring further research. In a comprehensive umbrella review, we synthesized the various categories and types of the used technology interventions, discussed their effectiveness and limitations, and finally explored their potential and need for further research. Finally, we amalgamated all the evidence from the umbrella review and used Grading of Recommendations, Assessment, Development, and Evaluations (GRADE) to make recommendations for interventions targeting social connectedness. This review attempts to answer the following questions:

1. What technology interventions are used to influence social connectedness in older adults?
2. How effective are these technology interventions in improving social connectedness in older adults, and what aspects make them effective?

## Methods

This umbrella review followed the standardized procedures [12,26,27] of systematic reviews. The protocol followed the PRISMA (Preferred Reporting Items for Systematic Reviews and Meta-Analyses) systematic review protocol guidelines [28] and the Joanna Briggs Institute (JBI) methodology for umbrella reviews [12].

### Search Strategy

The search strategy involved controlled vocabulary searching; phrase searching; and applying Boolean logic, limits, and filters. A comprehensive systematic search of 4 databases (PsycINFO, PubMed, Embase, and MEDLINE) was conducted between February 2020 and March 2022. The reference lists were also examined for additional reviews. The following search terms were used: “ageing,” “aging,” “older adults,” “reviews,” “2000-22,” and synonyms for “social isolation and loneliness,” “social connectedness,” and “technology interventions.” As an example, Textbox 1 shows the search terms and search strategy applied to the PubMed database. Search terms can be found in Multimedia Appendix 1.

**PubMed database search strategy (October 15, 2021; 176 results).**

**Search terms used**

- *SU (technology or computer or Internet) and TI (review or meta-analysis or metasynthesis) and SU (older OR aging OR aging OR aged OR elderly OR senior) and (social isolation OR loneliness OR social connectedness)*

**Search strategy applied**

- Limiters—Published Date: 20 000 101-20 211 231; Language: English; Publication Type: Academic Journal; English Language; Language: English; Year of Publication: 2000-22; Publication Year: 2000-22; Publication Type: Peer Reviewed Journal; English; Language: English; Exclude Dissertations Search modes—Boolean or Phrase Sort by best Match

### Inclusion and Exclusion Criteria

The inclusion criteria were formulated using the population, intervention, comparison or context, outcomes, and study schema [29,30]. Table 1 describes the inclusion criteria under which the studies were selected for this review.

**Table 1:** Inclusion and exclusion criteria.

PICOS^a^ framework	Inclusion criteria and reasons	Exclusion criteria and reasons
P—participants	Persons aged >50 years, who are living in community or residential settings with no major neurocognitive impairments	Participants aged <50 years
I—interventions	Interventions using any form of information and communications technology, smart communication devices, internet-based communication systems, information systems, video games, technological devices, and robots or technological pathways allowing for social interaction. These interventions must be specifically targeted at impacting or improving social connectedness in older adults	Nontechnology interventions, smart devices for home, or telehealth technologies not designed to impact social connectedness (eg, diabetes-measuring devices)
C—context	Community settings, independent living, and participants in nursing and care homes	Hospital settings, mental and physical illnesses, and disease or illness-specific cases
O—outcomes	Quantitative or qualitative outcome data or results focusing on social isolation or loneliness or social connectedness	Reviews lacking descriptions of outcome data
S—study	Review articles of any type using a systematic, qualitative, or quantitative method, including narrative, quantitative, and qualitative comparative studies. Articles must describe a clear intervention and include qualitative and quantitative comparative studies	Reviews with no technology intervention, no clear outcomes, or no systematic review processes. Reviews earlier than 2005 were not included because technology interventions before this time would not be directly comparable with ones of the present day

^a^PICOS: population, intervention, comparison or context, outcomes, and study.

### Selection Process

The abstracts and titles of all potentially relevant articles were screened. Full texts were then evaluated, and duplicates were removed. Uncertainties were discussed among the research team members to reach a consensus. Relevant data of the included articles were summarized in tables and checked for accuracy by a second investigator (CH).

### Analysis

The data analysis was based on a thematic synthesis with an inductive, iterative process consisting of 3 main stages: (1) free line-by-line review of the results, synthesis tables, and discussion sections of the included papers; (2) organization of themes into related areas; and (3) the identification, development, and refinement of detailed descriptions of factors that impacted the effectiveness of technology interventions [31]. All measures used were specified, and the statistical results (if provided) were summarized. The technology types were listed along with their effectiveness, and the authors’ conclusions were also summarized.

### Quality Assessment

The methodological qualities of the reviews were assessed using the Revised Assessment of Multiple Systematic Reviews (R-AMSTAR) [32] quality rating tool for reviews. The 11-item R-AMSTAR includes 11 questions (Multimedia Appendix 2 [19,20,23,24,33-52]) whose scores are summed to give the overall quality score of a systematic review. The R-AMSTAR tool provides a quantifiable assessment of systematic reviews and a measurement of their methodological quality. The maximum possible score is 44.15. Any review scoring <22 was excluded as it lacked 1 or more critical R-AMSTAR definitions. For example, the review might not assess the scientific quality of the studies or might apply a poor method for combining study findings [53].

### Grading of Evidence

The overall certainty of the evidence was evaluated using the GRADE method, which analyzes the risk of bias (imprecision, inconsistency, indirectness, and publication bias) and assesses the quality of the included evidence, which we used to make recommendations [54]. Initially, we categorized the evidence based on the inclusion or exclusion of randomized controlled trials (RCTs), followed by the inclusion or exclusion of observational studies. We then considered whether the studies had serious limitations or important inconsistencies in the results, or whether uncertainty about the validity of the evidence (the extent to which the participants, interventions, and outcome measures are similar to those of interest) was warranted (Multimedia Appendix 2). Limitations in study quality found in the R-AMSTAR appraisal, important inconsistency of results, or uncertainty about the directness of the evidence lowered the grade of evidence. For instance, if all available studies have serious limitations, the grade will drop by a level, and if all studies have very serious limitations, the grade will drop by 2 levels. The quality of evidence is also reduced by imprecise or sparse data and an imprecise understanding of social concepts.

## Results

### Overview

**Figure 1:**
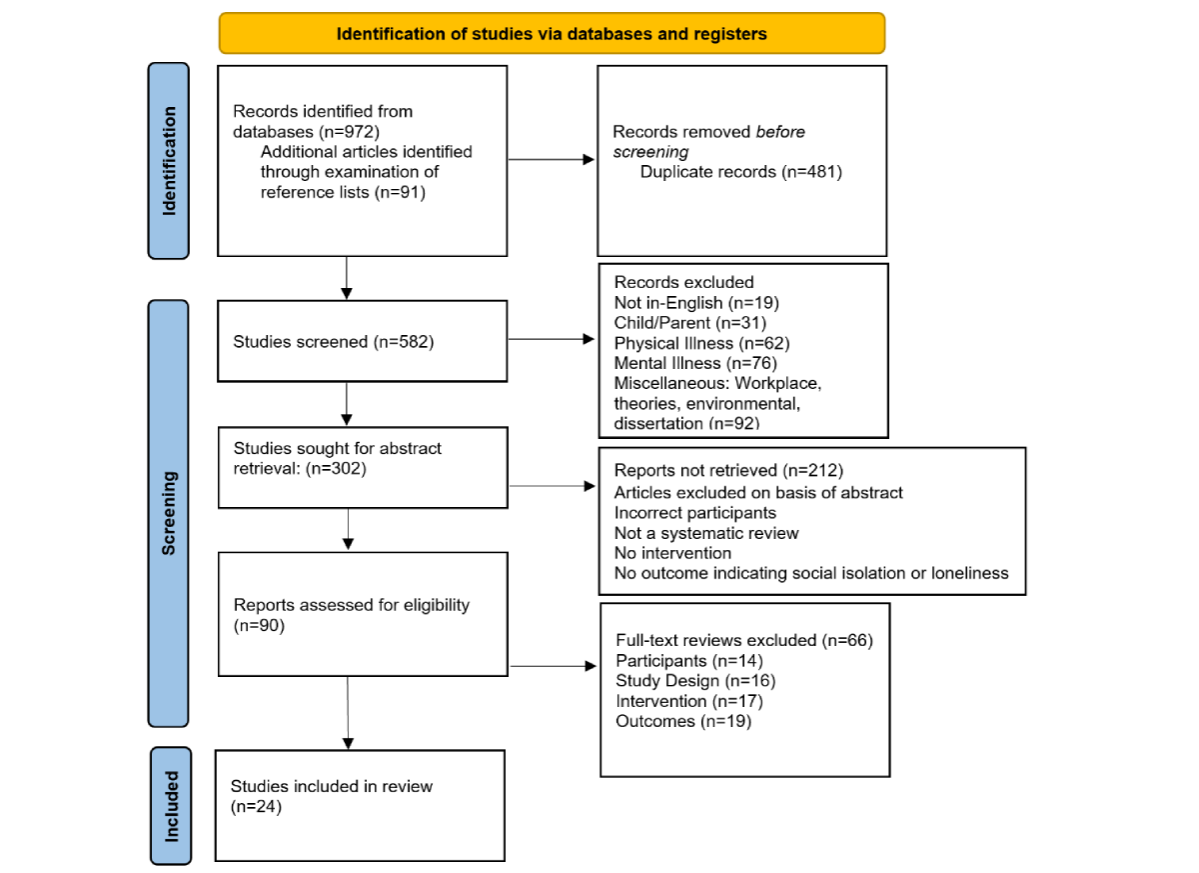
Flowchart of the literature search following PRISMA (Preferred Reporting Items for Systematic Reviews and Meta-Analyses).

The article elimination process is summarized as a flowchart in Figure 1. The initial search extracted 972 publications. Further, 91 articles were identified after checking the reference lists. After excluding duplicates and irrelevant publications, articles were screened using the population, intervention, comparison or context, outcomes, and study schema inclusion criteria (Table 1). The commonest reasons for exclusion were interventions targeted at specific mental and physical illnesses (138/972, 14.2%) and interventions not matching the prespecified definition (95/972, 9.8%). A total of 90 full-text reviews were further passed through a 3-step screening process (title, abstract, and full-text based) for eligibility and inclusion in the qualitative synthesis of this review. Finally, 24 reviews based on technology interventions were eligible for the synthesis.

### Quality Assessment

Among the 24 selected articles, 3 (12%) articles with R-AMSTAR scores <22 were excluded because they failed a priori systematic review processes (lacked clarity in scope or purpose, had a priori–defined participant population, had unclear outcomes of interest, lacked clarity on interventions, involved nonspecific subgroup analyses, and lacked meaningful hypotheses). The 21 remaining reviews were of moderate quality, with none meeting all of the R-AMSTAR criteria.

### Data Extraction

Data from the 21 reviews were extracted using a piloted, standardized data extraction form that captures and summarizes findings. As both technology interventions and extracted outcome data were heterogeneous, they were deemed inappropriate for a quantitative synthesis using meta-analytic techniques. Instead, a narrative synthesis summarizing the effectiveness of interventions was implemented. Under the methodological considerations of umbrella reviews, the results were reported descriptively in tabular form (Multimedia Appendix 3 [19,20,23,24,33-49]) along with their associated characteristics (number of articles, databases used, participants, types of interventions, study design, measures used, summary of results, authors’ conclusions, and summary and review methods). Multimedia Appendix 3 provides details of the 21 reviews in this study.

### Study Characteristics

The 21 selected reviews included 16 (76%) systematic reviews (reviews of evidence on a clearly formulated question and the use of systematic and explicit methods to identify, select, and critically appraise the relevant primary research), 2 (10%) integrative reviews (reviews that summarize past empirical or theoretical literature to provide a comprehensive understanding), 2 (10%) scoping reviews (preliminary assessments of the potential size and scope of the available research literature), and 2 (10%) meta-analyses (statistical analyses combining the results of multiple scientific studies). Most of the reviews covered the beneficial impact of technologies on loneliness, whereas others focused on social isolation, connectedness, and quality of life. General ICT was the most commonly applied intervention technology. The publication period was from 2005 to 2022, but 19 of the selected reviews were published within the last 7 years. Of the 21 reviews, 1 (5%) review focused on assistive technology for communication. Overall, 19% (4/21) of reviews focused on general interventions for social connectedness but examined technologies such as general ICT and videoconferencing, and 10% (2/21) of reviews focused on communication technologies for social connectedness in older adults. In all, 38% (8/21) of reviews investigated the impact of general internet and computer technologies on social isolation and loneliness. Of 21 reviews, 1 (5%) review examined the impact of smart technologies on social connectedness, and another (1/21, 5%) study reported the impact of health promotion technologies on social isolation and loneliness. In all, 10% (2/21) of reviews explored the ability of general ICT to improve the quality of life. Of 21 reviews, 1 (5%) review examined interventions to reduce social isolation and loneliness during the COVID-19 pandemic; 2 (10%) reviews focused on the impact of SNS on loneliness, another (1/21, 5%) examined interventions for preventing loneliness in nursing homes, and another (1/21, 5%) evaluated the benefits of telehealth in alleviating loneliness and increasing medication compliance. Here, telehealth was implemented through video health care professional visits to older adults. The 21 reviews covered a total of 326 underlying primary studies on technology interventions. It is worth pointing out that we were not able to confirm the presence of gray literature or studies that looked at technology interventions in the reviews.

The interventions discussed in the reviews were general ICT (11/21, 52%), videoconferencing (4/21, 19%), computer and internet training (3/21, 14%), telecare (2/21, 10%), SNS (2/21, 10%), and robotics (2/21, 10%). The reviews reported mixed results. Positive effects of ICT on loneliness were the most commonly reported, followed by the positive impacts of ICT on social isolation or connectedness. Reviewing data from the underlying primary studies in the reviews, the most effective intervention mode for social connectedness was identified as general ICT, followed by videoconferencing and robotics (Table 2).

**Table 2:** Effectiveness versus ineffectiveness of different intervention modes on social connectedness, identified in the underlying primary studies of the review papers (n=321).

Study intervention	Effective, n (%)	Ineffective, n (%)
3D or augmented reality (N=1)	1 (100)	0 (0)
Video gaming (N=4)	3 (75)	1 (25)
Videoconferencing (N=14)	11 (78)	3 (22)
Robotics (N=22)	16 (73)	6 (27)
Telecare (N=34)	22 (65)	12 (35)
SNS^a^ (N=61)	30 (49)	31 (51)
Computer training (N=66)	39 (59)	27 (41)
ICT^b^ (N=119)	86 (72)	33 (28)

^a^SNS: social networking site.

^b^ICT: information and communications technology.

### Results From Systematic Reviews With Meta-analyses

Among the 21 selected reviews, only Choi et al [20] and Bornemann [33] performed meta-analyses of homogenous data (Multimedia Appendix 3). Choi et al [20] reported a significant pooled decrease in loneliness after implementing technology interventions (*Z*=2.085; *P*=.04). However, Bornemann [33] concluded a nonsignificant decrease in loneliness after reviewing 5 out of 7 studies included in the review by Choi et al [20] (*Z*=.44; *P*=.37)—that is, the same 5 studies yielded different pooled meta-analysis results in the 2 reviews. This divergence indicates potential biases in the analytic approaches; for instance, Bornemann [33] excluded some studies included in Choi et al [20], and some of their findings were inconsistent with the narrative conclusions of their included studies. Bornemann [33] questioned the validity of some of the data acquired by Choi et al [20]. Although this review does not cross-examine these findings, we clarified that a study included in Choi et al [20] should have been excluded, as it was not an ICT intervention study. We decided that although the statistical conclusions of Bornemenn [33] were correct, Choi et al [20] raised some valid points. Multimedia Appendix 4 gives the levels of certainty in the quality assessment of outcomes developed within the GRADE framework. Low-quality assessments in different categories are mainly attributable to the elements of the study design, poor study quality, inconsistency, and indirectness.

### Categories of Technology Interventions

Of the 21 studied reviews, 14 (67%) dealt with general ICT (which was a catch-all term defining a diverse set of technological tools and resources used to transmit, store, create, share, or exchange information), 4 (19%) with videoconferencing, 3 (14%) with computer and internet training, 2 (10%) with telecare, 2 (10%) with robotics, 2 (10%) with SNS, 3 (14%) with gaming, and 1 (5%) with 3D augmented reality (AR). Among the primary studies, general ICTs were the most commonly adopted interventions (with 119 studies), followed by computer training, SNS, telecare, and robotics (Table 3). Although some of these categories overlapped, we differentiated them as they were distinguished in the original reviews.

**Table 3:** Frequencies of intervention categories in the primary studies (N=321).

Primary studies found in review	Frequency, n (%)
3D or augmented reality	1 (0.3)
Video gaming	4 (1.2)
Videoconferencing	14 (4.4)
Robotics	22 (6.9)
Telecare	34 (10.6)
SNS^a^	61 (19)
Computer training	66 (20.6)
ICT^b^	119 (37.1)

^a^SNS: social networking site.

^b^ICT: information and communications technology.

### Outcome Measures Used

All the reviews reported large numbers and diverse outcome measures of primary studies. Besides constructs of social disconnectedness (loneliness, social support, social contact, number of confidants, social networks, social connectedness scales, social isolation, and social well-being), many studies assessed factors such as quality of life, self-esteem, stress, and depression. Although not directly related to social disconnectedness, these factors may affect or be affected by social disconnectedness and may be useful to include outcome measures alongside social connectedness. A minority of the reviews also reported outcome measures of empowerment.

When analyzing these quantitative primary studies, the reviews commonly applied validated tools, such as the University of California Los Angeles (UCLA) Loneliness Scale (or a modified version) and the De Jong Gierveld Scale [4]. The UCLA was the most tested dependent variable. Among various other measures were the Social Support Scale by Schuster and Hunter [34], Social and Emotional Loneliness Scale [55], and Multidimensional Scale of Perceived Social Support by Zimet et al [56]. Social connectedness was sometimes measured using the holistic Social Connectedness Scale by Lee and Robin [57], which is regarded as a comparatively reliable measure.

The definitions and uses of outcome measures differed across the reviews. A total of 62 outcome indicators of social connectedness were used in the primary studies. Most reviews did not report on the lack of intervention effects (including the absence of significance values); moreover, the primary studies adopted a mixture of validated and nonvalidated outcome measures, making such reporting difficult. Consequently, they could not conclude whether the primary studies had validatable statistically significant outcomes.

### Social Concepts Used

The social concepts used for determining outcomes varied in range and diversity. In many reviews, the source papers did not define social participation or social isolation but instead evaluated these factors as general or neighboring concepts [19,35-37]. Loneliness was evaluated more consistently than social participation and social isolation but was sometimes incorrectly interchanged with social isolation. Most studies assessed loneliness on standardized scales, notably the UCLA Loneliness Scale [35,36,38,39].

A few of the reviews highlighted that inconsistency and lack of specific definitions hindered the grouping and evaluation of their chosen papers [19,37,39]. Morris et al [39] described social connectivity as a multidimensional concept that is difficult to define, conceptualize, and measure. They elaborated that outcome measures, such as the UCLA Loneliness Scale and Perceived Social Support Scale, identify only single aspects of social connectedness.

Cattan et al [37] also noted a complex association among social isolation, loneliness, and living alone, which was difficult to describe in their reviewed studies. Rarely among the review studies, Cattan et al [37] attempted to distinguish living alone from social disconnectedness and suggested that living alone be measured independently as a concept of physical isolation.

Ibarra et al [40] correctly defined loneliness as “a subjective measure referring to the ‘unpleasant’ lack of and quality of social relationships.” By contrast, isolation is an objective measure referring to few or no social relationships, although their study clarified the difference between social isolation and loneliness.

Gardiner et al [36] and Williams et al [38] adopted the less frequently used concept of *social facilitation* for creating mechanisms through which older adults can interact with peers. From an alternative perspective, they measured the facilitation of social connections. The article by Williams et al [38] was especially relevant, as it examined interventions during the COVID-19 pandemic. Facilitation may lead to effective interventions that reduce social isolation and loneliness, without violating COVID-19 shielding and social distancing measures.

In conclusion, different definitions and measurements of loneliness, social isolation, and social connectedness have led to diverse findings and wide variations across and within disciplines, defying a coherent picture of the research. Although some of the more recent studies and reviews have addressed this heterogeneity, reliable and succinct findings will remain elusive without further investigations.

### Group Interventions Versus One-to-One

Many interventions implemented in the individual papers of the reviews were broadly divisible into group and one-to-one interventions. In general, group interventions were more frequently implemented than one-to-one interventions, although both types were effective [24,37,40,41]. Cattan et al [37], who reviewed 3 computer group interventions, reported that group interventions with educational and social activities are particularly effective.

The imbalance between the group and one-to-one interventions impairs comparisons between the 2 types and conclusions regarding their comparative successes. Nevertheless, some of the reviews pointed out the possible advantages and limitations of these intervention approaches. Poscia et al [41] noted that group interventions might beneficially create a sense of security and belonging, although the real effect of the intervention might be obscured by interactions among the group members. Individual interventions might create deeper, more personal bonds and boost confidence in social engagements. Ibarra et al [40] further observed that one-to-one interactions limited participants’ contact with family, friends, and acquaintances, whereas group interventions encouraged them to interact with new people and potentially expand their networks, thereby increasing their number of new social connections.

Overall, group interventions appear to improve social disconnectedness, but the insufficient number of one-to-one interventions prevents an objective comparison and firm conclusions of the best interaction type. However, the GRADE assessment of the quality of evidence suggested a very low advantage of group interventions over one-to-one interventions (Multimedia Appendix 4).

### Effectiveness of Technology Interventions as an Overarching Category

Technology interventions that enhance social connectedness include general ICT, video games, robotics, and the Personal Reminder Information Social Management system (a custom-designed experimental SNS for older adults). Less conclusive evidence exists for the beneficial effects of SNS [20,24,25,37,41,42].

Overall, technologies appear to positively affect loneliness, social isolation, and other psychosocial aspects of older adults’ lives. Khosravi et al [42] examined 8 technology types and found that most technologies, in some formats, can increase social connectedness in older adults.

When technologies were intended to strengthen existing connections, their positive impacts on loneliness and social isolation were more consistent [24,40,41]. Ibarra et al [40] found that technologies are fundamental to long-distance interaction and are thereby necessary for expanding social networks, improving existing ties, and increasing social connectedness. However, they noted that how technology is availed, the limitations and opportunities of technology, and their effects on the success of the intervention are all unclear. Some reviews [20,35,43,44] included a psychosocial outcome of interest, such as social isolation, life satisfaction, loneliness, or depression. It was found that interventions significantly reduce loneliness but are ineffective against depression [35,43,45]. Damant et al [45] found a significant correlation between internet use and depression, suggesting that although the literature reports a significant correlation between loneliness and depression, technology can exert divergent impacts on these 2 psychosocial variables. However, Khosravi and Ghapanchi [43] reported that technology interventions can potentially reduce depression through engagement in social interaction, hinting that social isolation impacts more strongly on depression than does technology.

Choi and Lee [58] presented a detailed statistical evaluation of 8 RCT studies investigating the impacts of various technology interventions on loneliness. They found a statistically significant decrease in loneliness in the intervention group compared with the control and usual care groups (*P*=.07 and *P*<.001, respectively). However, there were no statistically significant differences in loneliness among the members of the intervention groups before and after the intervention (*P*>.05).

Individual reviews reported less conclusive outcomes of the overall technology use. The results of Morris et al [39] ranged from positive to no impact on loneliness, and Damant et al [45] noted a negative association between “social involvement and participation” and older adults’ use of technology, thereby indicating that the more socially involved people were, the less they tended to use technology. They found that high internet use was associated with high levels of loneliness. Interestingly, Chen and Schulz [35] found a positive effect of technology on social connectedness, this impact usually diminished in studies spanning >6 months. The time frame of studies investigating the effectiveness of technology was also a recurrent theme in other studies. The diminished effect is potentially linked to fatigue from using the intervention or inconsistency in the study approach over time.

Specifically, the following technology interventions appear to reduce social isolation but lack rigorous statistical support for a positive effect: robotics, telecare, and SNS [34,36,42,45].

Overall, 86% (18/21) of reviews examined the impact of technology intervention on loneliness. The reviews covered 324 primary studies involving 66,565 participants. Of the 18 reviews, 15 (83%) reported a positive effect of technology on loneliness; the remaining 3 (16%) studies found a 0 or negative effect. From the reviews, it can be concluded that technology interventions exert an overall positive influence on social isolation and loneliness (social disconnectedness), but their effectiveness depends on the design of the study. Longer training times, shorter study durations, and facilitation of existing relationships tended to increase the effectiveness of the intervention. The quality of evidence supporting the effectiveness of technology interventions on social connectedness (GRADE assessment) was moderate to low.

### General ICT

This section explores the findings of general ICT interventions reported in the reviews. General ICT is an umbrella term for generic technology devices, services, applications, and internet platforms [59]. ICT includes internet-based networks, mobile phones, computers, tablets, and any software requiring an internet connection. Interventions in this category include interactions via internet use (eg, discussions and forums), emails, video chats and conferencing, SNS, virtual spaces, classrooms, and messaging services. Some reviews mentioned systems tailored for older adults, such as the customized touch screen video-chat system described by Ibarra et al [40]. Computers with a mouse and keyboard as input devices were preferred, closely followed by tablets and mobile phones (the latter appeared as the most popular device in recent reviews). Other interventions used customized television sets and touch screen computers. Khosravi et al [42] and Khosravi and Ghapanchi [43] reported studies on Personal Reminder Information Social Management (a customized social networking platform). In most of the reviews, general ICTs were regarded as a single category, although videoconferencing and SNS were often placed in separate subcategories.

Many of the reviewed studies found that ICT interventions not only significantly reduce loneliness but also exert a positive impact on other aspects of social isolation, providing social support and connectedness, communication with family and friends, and ICT-accessible information sources [19,20,35,42,43]. Some reviews hinted that ICT facilitates the acquisition of information through the internet, either through interactions with other people or through finding relevant information on the web, which helps reduce loneliness [35,38,60]. Indeed, Morris et al [39] found that social connectedness especially benefits from technologies with web-based programs incorporating items such as health information, support groups, chat rooms, or discussion boards.

Damant et al [45] alone reported on studies with less promising results. In a study, only a small number of older adults maintained contact with their families via the internet. These participants were reluctant users with the sole purpose of keeping in touch with their grandchildren. In another study, they found no signiﬁcant correlation between internet or email use and contact with family and other people. Both studies revealed no signiﬁcant correlation between computer use or training and loneliness. Some of the studies reviewed by Damant et al [45] reported exacerbated loneliness through ICT use. It appears that ICT can positively reinforce existing social networks but has a limited impact on building new ones.

Only 2 reviews provided a homogenous meta-analysis. Both reviews reported positive impacts of general ICTs on social disconnectedness. In total, these reviews included 119 primary studies: 86 reporting a positive impact on social isolation or loneliness and 33 reporting unclear results or no impact. The studies agreed that increasing the frequency of general ICT use enhances social connectedness, improving the ease with which older adults can interact and maintain contact with others, thus reinforcing social connections with friends and family. The evidence that frequent ICT use facilitates the creation of new relationships or contacts is much weaker, further supporting, in part, the conclusions of Damant et al [45].

Together, these results suggest that general ICT can facilitate established connections and might supplement or replace older communication methods. Its role in establishing new connections is uncertain. Our results suggest that when considering ICT interventions (at least for older adults), it is important to distinguish between their ability to maintain relationships, potential ability to deepen relationships, and inability to help create new relationships. The GRADE strength of the ICT category, although only moderate, was the highest among the categories because a large number of primary studies, including RCTs, were reviewed in this category, and there was consensus and clarity on the outcome measures.

### Social Networking Sites

Although SNS is a subcategory of ICT, it warrants its own heading because 33% (7/21) of reviews discussed separate finding on SNS. The reviews gave mixed results. Whereas some studies supported the use of SNS in reducing loneliness, a sizable number showed no impact or even an increase in loneliness after SNS use [19,42,46]. Both Chen and Schultz [35] and Wiwatkunupakarn et al [46], who reviewed high-quality RCT studies on the use of SNS, reported inconclusive impacts of SNS on loneliness. They found some support for sites such as Facebook, which provides games that can be played with others over a network, thus fostering social interaction and alleviating loneliness. The mixed findings in these reviews might be explained as follows: although older adults embraced the use of SNS to support their social relationships and help them overcome loneliness, they did not regard these sites as a replacement for face-to-face contact. Participants preferred to use SNS for searching for and disseminating information rather than socializing. Morris et al [39] reported positive effects of smart technologies similar to SNS, especially when they incorporated health information, support groups, chat rooms, or discussion boards. Their findings support a role of SNS in knowledge-seeking and support-acquisition scenarios, with consequent impact on loneliness.

These findings may partly depend on the type of SNS, as different types of SNS support different features. For example, Facebook may promote socialization more effectively than YouTube, whereas YouTube may better facilitate explicit knowledge acquisition and information transfer than Facebook. Ibarra et al [40] discovered that participants favored off-the-shelf solutions, such as Facebook and About-My-Age (an SNS for older adults). Users of these sites commented on their decreased loneliness and easy control of the sites. The sheer volume of users on these platforms might assist older adults in finding relevant information, including information on how to use the platforms, thus creating a positive feedback loop.

On the downside, SNS use raises several concerns: privacy, lack of perceived usefulness, and possibly demographic factors [19,47]. Newman et al [47] noted an interesting connection between educational attainment and SNS use: SNS users tended to be White, employed, educated, and married. They also found attitude differences toward technology use among sociodemographic groups based on gender (women) and age (older people).

Overall, 61 primary studies examining SNS were found in the reviews: 31 reporting positive impacts of SNS on social isolation and loneliness and 30 reporting unclear or no impacts of SNS. Therefore, the effectiveness of SNS is inconclusive. The results suggest that older users can obtain support, acquire knowledge, and maintain their existing relationships through SNS. In terms of combating social disconnectedness and establishing new relationships, SNSs are less effective and can be detrimental at times. However, the effectiveness of SNS in developing new relationships, fostering and maintaining existing ones, and acquiring knowledge and support has not been explored in depth, and the idiosyncrasies of SNSs must be unraveled in further research. The strength of evidence (GRADE assessment) of the reviews in this category is low because of indirectness, missing information, and publication bias.

### Videoconferencing

Overall, videoconferencing appeared to exert a positive impact on loneliness and social connectedness. The visual aspect of this intervention seemed especially appealing to older adults [24,34-36,38,40,44,49]. In total, 3 reviews reported on videoconferencing between family members and their established contacts. All reviews described a statistically significant reduction in loneliness [39,41,45]; however, videoconferencing was more effective in facilitating established connections than in building new ones. Moreover, videoconferencing showed a weak impact on information gathering. For instance, Chen and Schultz [35] reported that videoconferencing did not significantly provide informational support (information communication for problem-solving assistance) or instrumental support (tangible goods, services, and aid), which may improve social connectedness [35]. Ibarra et al [40] mentioned 1 study in which Skype used for educational purposes did not change participants’ loneliness levels and another study in which Skype combined with computer training better reduced loneliness levels than did Skype alone. These reviews suggest that videoconferencing is effective for maintaining established connections, such as those with family members, but is less effective for other purposes, such as education and information seeking, which may indirectly impact social connectedness.

Gardiner et al [36] and Ibarra et al [40] mentioned the importance of appropriate hardware and design in videoconferencing. They reported that technical, financial, and design issues are potential barriers to the wider uptake of this technology.

When used in health support, videoconferencing yields mixed results. The intervention often decreases the loneliness and social isolation of residents in care and nursing homes, but a few studies have found no difference from the baseline [34,35,43]. More clearly, participants in these settings benefit from videoconferencing contact between family and friends, with beneficial effects on loneliness. Interestingly, Husebø and Storm [48] found that virtual visits by clinicians reduced the social isolation of residents in care homes, suggesting that videoconferencing can enhance the perception of independence by providing easy access to services. In general, videoconferencing appears to reduce loneliness in residential, nursing, and clinical care settings, although the specific aspects of the intervention that ensure its success have not been elucidated.

Overall, 14 primary studies in this subcategory were found in the reviews. Of these studies, 11 reported a positive impact on social isolation or loneliness. Owing to reviews such as by Schuster and Hunter [34], with clear outcomes and the inclusion of RCTs, the GRADE strength of evidence in this subcategory was moderate to low. The use of standardized outcome measures would have strengthened the GRADE rating.

### Mobile and Instant Messaging

Among the studied reviews, only Ibarra et al [40] alone described studies on MIMs such as WhatsApp and Line (messaging services). In 1 study, WhatsApp was used more extensively than email by relatives; however, a lack of responses can increase the perception of loneliness. Ibarra et al [40] hinted that as WhatsApp and similar applications are easy to use and allow the sharing of pictures, they exert a positive impact on social disconnectedness. However, the evidence was insufficient for concluding the impact of MIMs on social connectedness and loneliness; moreover, the few primary studies suggest that MIM explorations are only emerging at this stage. Given the lack of information found in the reviews, the GRADE strength of the evidence in this category was very low.

### Computer and Internet Training

In total, 13 reviews evaluated the impact of computer and internet training on various guises. All reviews found a positive impact of these interventions on social connectedness and loneliness [20,24,36,39,41,43,45]. In 4 of these reviews, loneliness reduction was found by the authors to be statistically significant [39,41,45]. However, all these studies investigated group training, suggesting that positive impacts were contributed (at least partly) by interaction with others in the group. Indeed, Damant et al [45] found a study in which group training increased the perceived support of friends and another study in which loneliness levels were reduced when email and web-based forums formed part of the training regime.

Mixed results were also obtained for this category. Baker et al [19] reviewed 2 studies on ICT training, 1 finding no correlation between the training and social connection and the other concluding that ICT training can enhance social networks. Although the authors did not elaborate on this discrepancy, the very different time frames of the 2 studies (12 months vs 8 weeks) may have affected the results. Indeed, whenever mixed results were found, the training time appeared to be a contributing factor, with shorter training times more likely to yield inconclusive results [24,36,42]. Furthermore, Choi and Lee [58] reported that in most studies, older adults enjoyed using technology and significantly increased their frequency of use, suggesting that minimal training was required.

Unusually, among the reviews, Williams et al [38] found that overall computer training produced no effect on social isolation. Overall, ICT training showed a higher ability to reduce loneliness in longer-duration studies than in shorter-duration studies.

As some reviews did not differentiate between the impacts of training and subsequent use, any assumptions would be dubious. Morris et al [39] noted a combined result, in which interactive web-based programs, discussion forums, and training mainly enhanced social connectedness; only 1 study reported inconclusive results. The effect of training was often confounded with the effect of the mechanism (such as group-based training), making it hard to differentiate and properly evaluate whether computer training on its own was having an effect. The GRADE strength of the evidence was low, emphasizing the need for assessing the full potential of computer training in social connectedness.

### Telecare

Telecare was among the less frequent interventions in the review studies, but when included, it appeared to reduce social isolation and loneliness [42,48,49]. Husebø and Storm [48] comprehensively investigated telecare services for older adults. After reviewing 12 primary studies covering this area, they found that virtual visits by clinicians can reduce social isolation and loneliness in older adults compared with no contact. Other benefits included self-management of medication and self-care, which can postpone admission to long-term care or substantial in-home care. In all areas, telecare both directly and indirectly affected participants’ perceived social isolation and loneliness. In 4 of the studies, older adults interacted with others experiencing similar issues. These interactions were highly valued and enabled the development of deeply empathetic connections [59]. By contrast, Damant et al [45] found no conclusive evidence of enhanced social connectedness among older adults using videoconferencing (14 studies).

Although none of the authors described the key features of successful telecare interventions, an emergent theme from successful primary studies was a high frequency of contacts. Interventions designed for regular and frequent contact were apparently more successful than interventions delivered on demand (eg, when a resident needed clinical attention). Overall, 34 primary studies in the analyzed reviews covered this category. The impact of telecare on social connectedness was inconclusive, and uncertainty was further increased by the poor reporting of the results. Consequently, the GRADE strength of evidence in this area was very low.

### Robotics

Robotics is a cutting-edge field and was mentioned in only 6 reviews. Some studies found that a pet robot provides the same level of benefit as animal-assisted therapy, which is known to reduce loneliness and social isolation [35,36,42]. Ibarra et al [40] mentioned that older adults feel embarrassed when conversing with a virtual pet, although this discomfort might have been exacerbated by audio problems and latency in messages. Choi and Lee [58] provided an excellent systematic review covering animal robots, humanoid robots, and mobile robots. They identified a notable development trend in robotic interventions from simpler animal robots to complex, multifaceted web-based social platforms that offer emotional support and promote social participation, cognition, physical activity, nutrition, and sleep. In most of their examined studies, robotic interventions decreased loneliness and social isolation. Although no other study has looked at the impact of virtual pets on loneliness, this seems to be a promising area that needs further research, with the potential of virtual or robotic pets offering a distinct advantage of social affordance compared with animal-assisted interventions.

Khosravi et al [42] and Antunes et al [44] examined conversational agents designed for companionship and video communication, enabling older adults to connect with family members and friends and offering “talk therapy.” Overall, these agents improved social interaction and reduced the loneliness of participants. With the ongoing development of pseudo–artificial intelligence (AI) technology and the advent of voice-assisted agents, such as Alexa and Siri, conversational agents are promising solutions and need to be further explored.

Khosravi and Ghapanchi [43] concluded that robotic technologies increase the perception of being socially connected and hence, exert a positive impact on social and emotional well-being. However, the perception of not being socially isolated differs from the actual reduction in social isolation, which depends on real person connections. On the adapted effectiveness scale, robotic technologies scored 1.8 out of 3.0.

Although these reviews indicate that social connectedness can be increased through robotics, this category is still new, and further studies on AI conversational agents and other robotic interventions are required. Therefore, the GRADE strength of evidence in this category is moderate to low.

### Gaming

According to Khosravi et al [42], Video gaming devices such as Wii, which capture natural physical activities, achieve a greater reduction in loneliness and better social interaction than typical video games. Chen and Schultz [35] and Williams et al [38] found that Wii strengthens social interaction and reduces loneliness; however, web-based gaming was outside the scope of these studies. Choi and Lee [58] reported 3 studies in which video games and exercises were combined into an exercise game, enabling communication with others. This game reportedly reduces loneliness during exercise. However, the GRADE confidence in the effect of gaming is very low because solid evidence is lacking.

### 3D and AR

Similar to robotics, 3D environments have been newly introduced as a loneliness-reduction intervention technique and are rarely reported. Khosravi et al [42] reported that most studies on 3D environments included a small number of participants, suggesting a need for further research. Although the underlying studies reported a positive impact of 3D environments on loneliness, the weak methodology and reporting of findings cast doubt on their validity. This category has been underexplored and requires further research. Current developments in 3D worlds, Facebook’s foray into Metaverse, and AR developments by prominent companies such as Google and Microsoft should accelerate the design of 3D interventions for older adults. Owing to a lack of evidence, the GRADE confidence in the effects of 3D environments and AR is very low.

### Usability Impact on Effectiveness of Technology

There were few reviews that examined the usability of technology and its impact on the effectiveness of interventions. Some reviews identified a link between usability and acceptance of technology; more accessible devices were distinctly more likely to be embraced by users than less accessible devices [19,40,48,58]. Even when usability was not a formal outcome, the studies observed participants’ initial feelings of uncertainty and fear of using technology. These trepidations were overcome with time, familiarity, and sufficient training [19,40]. Ibarra et al [40] reported that touch screen computers were especially effective in reducing loneliness and social isolation, highlighting the importance of an easily accessible system or interface. Husebø and Storm [48] noted that when introducing technology to older adults, a usable and simple design that considers the likely interactions of older adults with technology is essential. Choi and Lee [58] identified 6 studies in which the use of and attachment to ICT interventions increased over time along with the average density of social networks.

However, systematic reviews typically neglect the human-computer interaction components of intervention technology. Moreover, standardized measures of usability (eg, the System Usability Scale) for intervention studies have not been defined [19,40]. The use and adoption of technology by older adults largely depends on the learning ability of the individual and the perceived difficulty of use. To ensure that technology can effectively reduce loneliness in older adults, these potential barriers should be examined appropriately.

Overall, the reported studies showed that whether technology can reduce loneliness depends on its usability. An intervention perceived as difficult to use by older adults cannot be effective. This aspect must be further investigated to improve the success of technology interventions.

Owing to a lack of evidence, the GRADE confidence in the effect of usability on the success of intervention technologies is very low.

### Summary Recommendations

On the basis of the results, Table 4 summarizes the key recommendations extracted for technology interventions targeting social isolation, connectedness, and loneliness.

We have also summarized the key recommendations for study design targeting social isolation, connectedness, and loneliness in Table 5.

**Table 4:** Summary of key recommendations for technology interventions.

Category	Key recommendations	Certainty of evidence
General ICT^a^	Simple technology interventions can be more successful than complex ones. Usability is a potentially important outcome. ICT is not recommended for increasing either the quantity or quality of communications or helping to establish new relationships. It is recommended for maintaining and enhancing existing relationships and access to services (such as health-related services).	Moderate
SNS^b^	SNS is not recommended as an intervention for loneliness and isolation as SNS use has often been shown to worsen loneliness. SNS is useful in knowledge and support acquisition scenarios, which can themselves reduce loneliness. Research shows that SNSs are generally more successful in these scenarios than in making new connections. Privacy is an important concern among older adults and needs to be considered when designing an intervention. Usability is potentially a very important theme and needs to be factored into the study design.	Low
Videoconferencing	Videoconferencing reduces loneliness by providing social support and improving the existing conditions in health care–type situations. Financial investment (eg, cost of computer hardware) needs to be considered when planning a videoconferencing intervention.	Moderate low
MIM^c^	MIM is recommended for rapid deployment as it is easy to use, and applications such as WhatsApp additionally allow the sharing of pictures, which can improve social connectedness. MIM can replace email, but designers must be wary because any lack of responses can increase the perception of loneliness.	Very low
Computer and internet training	Longer training periods are recommended with shorter-duration studies (as highlighted above) as they have been the most effective. For reducing loneliness, group-based training is more effective than one-to-one training. The study design should reflect whether the training or use of the intervention causes reduction in loneliness. RCTs^d^ are particularly important in the study design as they determine precise effect sizes.	Low
Telecare	Frequency of contact combined with telecare solution influences the success of an intervention. Interventions designed for regular frequent contact are more successful than interventions delivered on-demand; for example, when a resident needs clinical attention. Videoconferencing groups such as group counseling can help to reduce feelings of anxiety, isolation, and loneliness and provide emotional and social support; however, designers must understand that some participants do not immediately feel at ease with others, especially in a group setting.	Very low
Robotics	Pet robots can provide the same advantages as animal-assisted therapy in reducing loneliness and social isolation; study designs can mimic previous studies in this area. Conversational agents provide companionship through social interaction, enabling older adults to connect with family members and friends (social presence). These agents can be effective and are recommended for intervention studies. RCTs are recommended in the study design of robotic interactions, especially as this area is understudied.	Moderate low
Gaming	Video gaming devices such as Wii, which capture natural physical activities, are recommended as they reduce loneliness and provide better social interactions than typical video games.	Very low
3D and augmented reality	Too few of the existing studies provide robust recommendations, and further longitudinal and cross-sectional RCT studies are needed in this area.	Very low

^a^ICT: information and communications technology.

^b^SNS: social networking site.

^c^MIM: mobile instant messaging.

^d^RCT: randomized controlled trial.

**Table 5:** Summary of key recommendations for technology interventions.

Category	Key recommendations	Certainty of evidence
Group vs one-to-one	Studies should be designed as group-based interventions, as they appear to better facilitate social connectedness than one-to-one interventions.	Very low
Effectiveness of technology interventions	Certain types of technologies (information and communications technology and videoconferencing) are particularly suitable as interventions for social isolation and loneliness. For best results, studies should be designed to strengthen existing bonds, especially the connections between family members (eg, grandchildren).	Moderate low
Frequency of use	Frequency of use is encouraged (greater use increases the effect size).	Very low
Training	Training, especially in the use of technology, is encouraged as it improves the success of the study.	Very low
Duration	Shorter-duration studies are recommended (shorter studies achieve better results than longer-duration studies).	Low
Outcome measures	The impact of intervention is stronger on social isolation than on loneliness, and studies should be designed to look further on how to impact loneliness. Use of standardized measures such as University of California Los Angeles Loneliness Scale and the Lubben Social Network Scale is recommended.	Low
Mechanisms	Mechanisms by which interventions reduce social isolation through the design of studies, including the gaining of social support, engagement in activities of interest, the making of new connections, and search for new information, should be clearly defined at the outset.	Very low
Usability	Intervention studies should adopt standard measures of usability (eg, System Usability Scale) because the adoption of technology by older adults largely depends on learnability and perceived difficulty of use. These barriers often prevent technology from reducing loneliness in older adults.	Very low

## Discussion

### Principal Findings

This umbrella review, as highlighted in the analyzed reviews, found that different studies adopted a vast diversity of outcome measures and nonstandard definitions of loneliness and isolation [20,33,35,39,42], and therefore, heterogeneity, lack of clarity, and lack of consistency across reviews have influenced the interpretations of their findings. The strengths of the evidence for effectiveness ranged from very low (robotics, telecare, 3D or AR, and video games) to moderate (ICT). These low ratings were attributed to the poor overall quality of evidence, study design, and outcomes. However, our umbrella review showed that despite the heterogeneous quality and diverse scope of existing reviews, which prohibit the drawing of generalizable conclusions, technology can effectively target social disconnectedness in older adults [61,62].

An umbrella review following the JBI methodology [12,26] was warranted because the types of reviews, levels of evidence, and outcomes of different reviews range widely in quality, from meta-analyses to qualitative syntheses, and the availability of a wide range of reviews allows our umbrella review to comprehensively consolidate the current state of evidence on interventions for social connectedness. As highlighted in the analyzed reviews, different studies adopted a vast diversity of outcome measures and nonstandard definitions of loneliness and isolation [20,33,35,39,42], and therefore, heterogeneity, lack of clarity, and lack of consistency across reviews have influenced the interpretations of their findings. Many of the review authors included social isolation and loneliness interchangeably when selecting their intervention studies, failing to recognize that each condition is a component of social disconnectedness. This confusion weakens the recognition of differing results, as loneliness is generally more resistant to interventions than social isolation. Although some loneliness measures (eg, UCLA and De Jong Gierveld Scale) have been regularly adopted, the Lubben Social Connectedness Scale was applied in only 9 of the primary studies. This scale, which assesses an individual’s psychological sense of belonging, might better reflect the interaction among different dimensions of social connectedness than commonly adopted measures [39,60]. Most of the primary studies developed their measures or used less common measures, such as the Self Anchoring Scale, Social Network Structure, Social Supportive Behavioral Scale, and Social Connectedness Index.

The designs and qualities of the reviewed primary studies varied widely. Several reviews included RCTs and pilot, qualitative, and quantitative studies. In addition, the studies reviewed by Khosravi et al [42] were conducted across the health domain. The primary studies in each review are typically nonoverlapped, indicating that the reviewers’ searches did not capture all relevant studies and sometimes omitted important studies and assessments of bias risk.

The findings of many underlying primary studies in the reviews were compromised by poor study designs, leading to conflicting information. For example, when reviewing the effects of computer and internet training on loneliness, Chen and Schulz [35] reached an inconclusive verdict, Choi et al [20] reported a significant impact of the intervention, and Bornemann [33] demonstrated no significant effect of the intervention. Moreover, the effect size calculated by Bornemann [33] differed from the more accurate calculation by Choi et al [20], although both reviews shared 5 primary studies in their meta-analyses.

The reviewers generally agreed on the effectiveness of group-based interventions. Reviews examining the designs of the reviewed studies noted group-based interventions yielded positive effects on social disconnectedness [24,36,37,40,41]. The different effects of group interventions can be attributed to the social interaction value of being in a group rather than the actual intervention [36,37]. When the intervention was delivered over a longer duration, the effect of the group activity diminished over time, and the intervention became less effective. Interventions with a participatory, productive, and collaborative focus [36], especially educational [37], appeared to realize an effective group-based intervention.

The reviews varied in scope, from assessments of the effectiveness of interventions, such as videoconferencing, to overviews of studies published in the field. The inclusion criteria and quality assessments of the primary studies also differed among the reviews, diminishing confidence in their findings. Our study confirmed a low quality of evidence in this field, whereas improved technology interventions for older adults are increasingly demanded by both policymakers and health professionals. Although the existing guidelines can encourage standardization of systematic reviews, these guidelines were largely ignored by researchers; accordingly, the strength of the reviews is diminished, which in turn led to the quality of evidence GRADE scores also being generally low.

The scope of the reviews varied from a specific focus on the effectiveness of a targeted intervention (such as computer training) to an overview of the published studies in the field. The inclusion criteria for the primary studies and their quality assessment depended on tools used for rating rigor and bias. Such variations cast doubt on the conclusions of these reviews. This review confirms the lack of high-quality evidence in the field and highlights the failure to adhere to the existing guidelines. Standardization of systematic review reporting is expected to strengthen confidence in the review conclusions.

Unlike their younger counterparts, older adults often lack the skills, functional capacity, and accessibility to adopt digital technology [63], which has led to the so-called “digital divide” among populations. However, these expansive categories are not mutually exclusive to older adults. In resource-restricted settings, they also incorporate gender differences, age, economic status, cultural practices, and educational qualifications [63] and can play an important role in reducing the existing digital divide between younger and older adults. Most of the reviews did not adequately consider these differences, presuming a general dearth of resources for older adults. Also important are the usability and design of the intervention, which were notably absent in the primary studies. The individual circumstances of older adults (including finances, environment, and access to resources) may influence the success of interventions. When usability was examined (as in some reviews), it was done without the use of standardized usability measures, but usability did influence the effectiveness of the intervention; therefore, further exploration of this area is vitally important.

To improve the quality of results, interventions should be tailored to match the specific needs of older adults, and sufficient training should be provided for using the interventions. This tailoring requires the involvement or participation of participants in training in a variety of formats [24,41]. As usability issues can reduce the effectiveness and uptake of an intervention, neglecting usability as an outcome measure reduces confidence in a holistic discussion of the effectiveness of an intervention. Thus, the potential impact of technology on social connectedness in older adults requires further investigation.

### Comparison With Prior Work

Our umbrella review is one of the few works that have looked at technology interventions for social connectedness for loneliness, following a well-established systematic approach such as the JBI umbrella review method. In examining other works, we came across reviews that focused on interventions generally [64-67] as opposed to technology interventions, which we noted was a tendency to bundle technology interventions with other common interventions, such as cognitive enhancement work groups, adult day center attendance, gender-based social groups, activities such as befriending and mentoring programs, and animal-assisted therapy. The problem with this approach is that it dilutes attention away from the different types of technology interventions and how they individually affect social connectedness and compares them as a category to other types of interventions. The fallacy of this approach for technology interventions is that it only presents high-level evidence and comparisons of diverse interventions, preventing readers from understanding the deeper nuances that make certain technology interventions more successful than others. Indeed, many of reviews of reviews are scoping reviews [66,67], corresponding to a more expansive inclusion criterion, which limits more substantive findings about specific interventions and rather presents a broader scope of general findings. Our umbrella review, on the other hand, by focusing specifically on technology interventions, extends the understanding of specific technology interventions by reporting and rating the evidence. By doing so, we were able to identify current evidence gaps related to the understanding of the types of technologies, study design, and their impact on social connectedness, loneliness, and isolation. We were also able to identify weaknesses in the reviews and areas for future research.

### Strengths and Limitations

Most of the reviews demonstrated a need for stronger evidence on the effectiveness of technology interventions that reduce loneliness. Weak methodologies have limited the ability of reviews to establish conclusive remarks on their effectiveness [35,42]. Many outcome measures have greatly limited comparisons, which affected the interpretation of the results.

The present review may also have been biased by accepting only English-language publications. However, many of the shortcomings and limitations of this umbrella review stem from the underlying problems of the primary papers included in the reviews. Among the common shortcomings were small-scale implementations with small sample sizes, low levels of evidence, and short periods of assessment.

Another recurring limitation was the inconsistent definitions of social concepts. Social concepts such as loneliness, social isolation, and social connectedness were formally defined, but the authors did not use these definitions consistently; instead, they were often used interchangeably, inherently confounding measurements of these outcomes. The reviews were generally heterogeneous in focus (eg, addressing loneliness and depression) and discussed various interventions and syntheses of outcomes (eg, meta-analyses, qualitative reviews, and mixed methods). Accordingly, the present review interchanges the terms *social connectedness* and *social disconnectedness* to describe combinations of singular aspects such as social isolation and loneliness. Nevertheless, the methodology was the greatest limitation. Finally, the absence of gray literature in the reviews may have increased publication bias and led to the lack of inclusion of evidence for interventions that are not typically indexed in bibliographic databases. Future systematic reviews should consider including gray literature in the included studies.

The methodological limitations of the reviewed studies impaired the internal validity and usefulness of the reviews for technical and policy decision-making, as highlighted by the reviewers [20,24]. The reviews reported on diverse methodologies, including the use of nonstandardized outcome measures, which broaden the perspective but risk biasing the conclusions. Furthermore, as interventions vary widely in nature, direct comparisons are difficult, and the definitions of technology interventions are rather narrow in some studies [39].

The reviewed quantitative studies collected their data with questionnaires using scales developed for the study purpose. The reliability and validity of these nonstandardized scales are difficult to evaluate. Most reviews pointed out the suboptimal methodological quality of studies in this field, particularly the scarcity of RCTs (<28% of studies) and the dominance of quasi-experimental studies, which challenge the delivery of robust conclusions.

Therefore, the results of this review should be interpreted with caution.

### Suggestions for Future Research and Policy Implications

Various technology interventions in different formats offer many ways to engage older adults. However, usability was rarely discussed in the reviews and was not assessed as an outcome measure. Although the existing guidelines encourage the standardization of systematic reviews, they have not been followed with the required rigor. Equally, the underlying primary studies of the reviews failed to address causation in a rigorous study design, and their heterogeneity limited their generalizability. It appears that there is a need for more studies on the multidimensional impact of technology on social connectedness, along with the assessment of other measures that may be interacting with technology use (such as educational attainment, psychological resilience, and age-friendliness of environments). Robotics is a relatively new technology that has emerged to be promising, but there are very few studies in this domain. Research on mobile technology interventions for social isolation is also encouraged as mobile phone technology provides opportunities for increasing the uptake of technology interventions targeting loneliness in older adults. Our results on the grading of evidence revealed that the strength of evidence was generally low to very low, indicating that the efficacy of the interventions is unclear and that more rigorous research is needed.

Our review provides insights into strategies to reduce loneliness and isolation for older adults using technology interventions, with implications for future research, policy, and practice. Attention to social connections needs to be incorporated into existing preventative efforts for chronic diseases in older adults. Chronic illnesses develop slowly over decades. Since social connectedness is known to impact multiple mechanistic pathways in both the development and progression of disease, it warrants attention in primary, secondary, and tertiary prevention efforts. Given the lower economic costs of technology interventions for individuals, families, employers, and the broader health care system, we urge health care and health policy professionals to prioritize the investigation of technology interventions for social connections in prevention efforts.

### Conclusions

This umbrella review consolidates the state-of-the-art knowledge on the types of technology interventions that influence social connectedness in older adults and their effectiveness. The data were collected from the last 2 decades. Technology purportedly enables long-distance interactions, allowing older adults to become socially connected, obtain support, expand their social networks, and strengthen their existing ties. Some important themes that would improve the effectiveness of technical interventions for older adults emerged from the literature, namely group interventions, short-duration training and study programs, the use of general ICT, and videoconferencing. These implementations are more effective for maintaining existing connections than for building new ones. Certain technologies, such as robotics (including virtual pets), AI-based conversational agents, and MIMs, show promising potential but have been underexplored.

We attempted to determine which technology interventions can effectively improve social connectedness. The following conclusions emerged from our study. Reports on the effectiveness of computer and internet training on loneliness and social isolation provided mixed and inconclusive results. General ICT and internet-mediated communications were shown to reduce loneliness and social isolation in most studies, although the results apparently depend on the frequency of use and the time frame of the study, with shorter studies being more successful than longer ones. ICT interventions help socially isolated older adults through a range of mechanisms, including gaining social support, providing connections to the outside world, introducing new friends, and boosting self-confidence. All of these mechanisms must be studied hand in hand to gain a complete understanding of these processes. Finally, in our GRADE evaluation, most of the evidence was rated as moderate low to very low, reflecting methodological issues, the small number of RCTs, diverse outcome measures and definitions, and mixed results. Such low scores highlight the need for high-quality research in this area.

## Appendix

Multimedia Appendix 1 Search terms used in database.

Multimedia Appendix 2 Revised Assessment of Multiple Systematic Reviews quality ratings.

Multimedia Appendix 3 Characteristics of the included reviews.

Multimedia Appendix 4 Grading of Recommendations, Assessment, Development, and Evaluations—certainty of evidence.

## Abbreviations

AI: artificial intelligence
AR: augmented reality
GRADE: Grading of Recommendations, Assessment, Development, and Evaluations
ICT: information and communications technology
JBI: Joanna Briggs Institute
MIM: mobile instant messaging
PRISMA: Preferred Reporting Items for Systematic Reviews and Meta-Analyses
R-AMSTAR: Revised Assessment of Multiple Systematic Reviews
RCT: randomized controlled trial
SNS: social networking site
UCLA: University of California Los Angeles
