# Supplementary material for: Effectiveness of Technology Interventions in Addressing Social Isolation, Connectedness, and Loneliness in Older Adults: Systematic Umbrella Review
Source: JMIR Aging. 2022 Oct 24;5(4):e40125. doi: 10.2196/40125 (PMC9641519; doi:10.2196/40125)
Supplement: Multimedia Appendix 1 [file aging_v5i4e40125_app1.docx]

**Appendix 1 – Search Terms for Databases**

All searches were performed by First Author and checked by Second Author.

| PubMed | SU (technology or computer or Internet) and TI (review or meta-analysis or meta synthesis) and SU (older OR aging OR aging OR aged OR elderly OR senior) and (social isolation OR loneliness OR social connectedness) Limiters—Published Date: 20 000 101-20 211 231; Language: English; Publication Type: Academic Journal; English Language; Language: English; Year of Publication: 2000-21; Publication Year: 2000-21; Publication Type: Peer Reviewed Journal; English; Language: English; Exclude Dissertations Search modes—Boolean/Phrase Sort by best Match |
| --- | --- |
| PsychINFO | Search: (Aging OR Ageing [All fields] OR AB “older adults” or AB elderly) AND (AB technology OR Computers [All Fields] OR internet [All Fields] OR “information and communication technology” [All fields] OR “robot” [All Fields] OR “technology interventions” [All Fields] OR “social media” [All Fields]) AND ( AB loneliness OR loneliness [All fields] OR AB “social isolation” OR “social isolation” [All Fields] OR “social connectedness” [All Fields] ) AND ( AB intervention OR program [All Fields] OR evaluation [All Fields]) AND ( Systematic Review OR Scoping Review [All Fields] OR Review [All Fields]) 2000-2021, English |
| EMBASE | loneliness/  Loneliness.ti,ab.  (lonely or social isolation).ti,ab.  1 or 2 or 3  (technology* or digital* or technol* or sensor* or robot* or internet* or social media or smartphone* or smart phone* or online or ipad* or mobile phone* or computer* or electronic* or Web).ti,ab.  robotics/  Internet/  social media/  smartphone/  artificial intelligence/  bot/  computer/  mobile phone/  cell phone/  5 or 6 or 7 or 8 or 9 or 10 or 11 or 12  4 and 13  limit 14 to (English language)  limit 15 to yr=“2000 -Current” |
| MEDLINE | Search: ( MM Aged [MeSH term] OR MM “Aged, 55 and over”[MeSH term] OR TX “older adults” OR TX elderly OR Aging OR Ageing) AND ( MM technology [MeSH term] or technology [All Fields] OR MM “Technology Interventions” OR TX Computers OR MM internet [MeSH term] OR internet [All Fields] OR MM “computers, handheld” [MeSH term] OR MM “Information and communication technology” [MeSH term] OR Robot [All Fields] OR Mobile Technology [All Fields]) AND ( MM loneliness [MeSH term] OR loneliness [All Fields] OR MM “social isolation” [MeSH term] OR social isolation [All Fields] OR social connectedness [All Fields]) AND ( intervention [All Fields] OR program [All Fields] OR evaluation [All Fields]) AND (Systematic Review [All Fields] OR Review [All Fields]) 2000-2021, English |
