# Supplementary material for: Effectiveness of Technology Interventions in Addressing Social Isolation, Connectedness, and Loneliness in Older Adults: Systematic Umbrella Review
Source: JMIR Aging. 2022 Oct 24;5(4):e40125. doi: 10.2196/40125 (PMC9641519; doi:10.2196/40125)
Supplement: Multimedia Appendix 2 [file aging_v5i4e40125_app2.docx]

## **Appendix 2 –R-AMSTAR quality ratings**

**Table of R-AMSTAR ratings of the reviews**

| **Reviews** | | | | | | | | | | | | | | | | | | | | | | | | |
| --- | --- | --- | --- | --- | --- | --- | --- | --- | --- | --- | --- | --- | --- | --- | --- | --- | --- | --- | --- | --- | --- | --- | --- | --- |
| **Rating** | Antunes et al. | Baker et al. | Bornemann | Cattan et al. | Chen & Schultz | Choi et al. | Campus et al. | Cohen-Mansfield | Damant et al. | Kachouie et al. | Gardiner et al. | Husebø & Storm | Ibarra et al. | Khosravi et al. | Khsoravi and Ghapanchi | Morris et al. | Poscia et al. | Schuster and Hunter | Stojanovic | Williams et al. | Wiwatkunupakarn et al. | Esandi & Alfaro | Choi & Lee | Newman, Stoner, Spector |
| Was an ‘a priori’ design provided? | 4 | 4 | 4 | 4 | 3 | 3 | 2 | 3 | 4 | 2 | 4 | 4 | 4 | 4 | 4 | 3 | 4 | 4 | 4 | 2 | 2 | 1 | 2 | 2 |
| Was there duplicate study selection and data extraction? | 4 | 3 | 4 | 4 | 2 | 3 | 1 | 1 | 2 | 2 | 4 | 4 | 1 | 4 | 3 | 4 | 4 | 2 | 2 | 4 | 4 | 1 | 4 | 1 |
| Was a comprehensive literature search performed? | 3 | 3 | 4 | 4 | 4 | 3 | 4 | 4 | 4 | 2 | 4 | 4 | 2 | 4 | 3 | 4 | 4 | 3 | 3 | 4 | 4 | 3 | 4 | 4 |
| Was the status of publication (ie, gray literature) used as an inclusion criterion? | 2 | 1 | 4 | 4 | 2 | 3 | 2 | 3 | 3 | 2 | 4 | 2 | 2 | 4 | 2 | 3 | 4 | 3 | 4 | 2 | 3 | 1 | 4 | 4 |
| Was a list of studies (included and excluded) provided? | 2 | 2 | 2 | 2 | 2 | 2 | 2 | 2 | 2 | 2 | 2 | 2 | 2 | 2 | 2 | 2 | 2 | 2 | 2 | 3 | 2 | 1 | 3 | 4 |
| Were the characteristics of the included studies provided? | 4 | 2 | 4 | 4 | 3 | 4 | 3 | 4 | 4 | 2 | 3 | 4 | 4 | 4 | 4 | 4 | 4 | 4 | 4 | 3 | 4 | 1 | 4 | 4 |
| Was the scientific quality of the included studies assessed and documented? | 2 | 1 | 4 | 4 | 3 | 4 | 1 | 1 | 2 | 3 | 4 | 2 | 1 | 4 | 4 | 4 | 4 | 4 | 2 | 3 | 4 | 1 | 4 | 4 |
| Was the scientific quality of the included studies used appropriately in formulating conclusions? | 3 | 1 | 4 | 3 | 4 | 4 | 1 | 2 | 2 | 1 | 3 | 2 | 2 | 3 | 4 | 3 | 3 | 3 | 2 | 3 | 2 | 1 | 3 | 3 |
| Were the methods used to combine the findings of studies appropriate? | 3 | 3 | 4 | 3 | 3 | 4 | 1 | 3 | 4 | 1 | 3 | 3 | 3 | 3 | 3 | 3 | 3 | 3 | 3 | 2 | 1 | 1 | 2 | 3 |
| Was the likelihood of publication bias (a.k.a. “file drawer” effect) assessed? | 1 | 1 | 3 | 1 | 1 | 1 | 1 | 1 | 1 | 1 | 1 | 1 | 1 | 1 | 1 | 1 | 1 | 1 | 1 | 2 | 1 | 1 | 1 | 2 |
| Was the conflict of interest stated? | 2 | 2 | 3 | 2 | 2 | 2 | 1 | 1 | 3 | 2 | 3 | 3 | 3 | 1 | 1 | 2 | 3 | 3 | 2 | 3 | 3 | 1 | 3 | 3 |
| **TOTAL R- AMSTAR** | **30** | **23** | **40** | **35** | **29** | **33** | **19** | **25** | **31** | **20** | **35** | **31** | **25** | **34** | **31** | **33** | **36** | **32** | **29** | **31** | **30** | **13** | **34** | **34** |
| **Include** | **Y** | **Y** | **Y** | **Y** | **Y** | **Y** | **N** | **Y** | **Y** | **N** | **Y** | **Y** | **Y** | **Y** | **Y** | **Y** | **Y** | **Y** | **Y** | **Y** | **Y** | **N** | **Y** | **Y** |
