# Supplementary material for: Effectiveness of Technology Interventions in Addressing Social Isolation, Connectedness, and Loneliness in Older Adults: Systematic Umbrella Review
Source: JMIR Aging. 2022 Oct 24;5(4):e40125. doi: 10.2196/40125 (PMC9641519; doi:10.2196/40125)
Supplement: Multimedia Appendix 3 [file aging_v5i4e40125_app3.docx]

## **Appendix 3 –** **Characteristics of the Included Reviews**

| **Author/Year** | **Study Aim** | **Number of Articles and Databases Used** | **Participants** | **Types of Intervention** | **Study Design** | **Measures Used** | **Summary of Results** | **Authors Conclusions and Summary** | **Review Method** |
| --- | --- | --- | --- | --- | --- | --- | --- | --- | --- |
| Antunes at al. (2018)[49] | How assistive technology enhances communication for older adults | 18 studies (2 databases, 2001–2016) | 3–105 participants aged 9–98. Total of 533 participants. | Assistive technology for people with speech problems; Robots and videoconferencing systems, Information and communication technologies | Randomized Control Trial (RTC): 1  Quantitative: 5  Qualitative: 12 | UCLA and QOL (Quality of Life) | Only 3 studies related to ICT, all findings show a positive impact of assistive technology on loneliness | Reduced loneliness in older adults using videoconferencing for socializing and training on internet use | Systematic review |
| Schuster and Hunter (2019)[36] | Impact of video communication among nursing home residents | 5 studies (PubMed and EBSCOhost, 2002–2015) | 2–231 participants aged 60+ | Video communication | Quantitative: 4  Qualitative: 1 | UCLA scale, Geriatric Depression Scale, Mini-Mental State Examination (MMSE) | 4 out of 5 studies showed that video communication reduces loneliness. | Overall decrease in loneliness but no statistically significant results from the study using the UCLA scale | Scoping Review |
| Chen and Schultz (2016)]40] | Effect of ICT interventions on reducing social isolation in older adults | 25 studies (9 databases, 2002–2015) | 8–5203 participants aged 55–105 | 7 Computer/ Internet training Computer use 2 Videoconferencing/Skype 1 Gaming–Wii | RCT: 4  Mixed: 4  Qualitative: 8 | 2 DJGLS(De Jong Gierveld Loneliness Scale)  1 Self-Reported  5 UCLA-LS  1 Belief in External Control Scale  2 Perceived Control of Life  1 Self Anchoring Scale  1 Social Network Structure  1 Social Supportive Behavioral Scale | 15 out of 18 studies reported a positive impact of ICT on loneliness; 3 studies reported no impact | High attrition rate. ICT intervention uptake improved with training. Inconclusive result of impact on loneliness. Implication that ICT intervention is not suitable for everyone. | Systematic Review |
| Baker et al. (2018)[19] | Current use of technology to combat social isolation among the elderly | 36 studies (5 databases, 2006–2017) | 8–388 participants | Touch-screen and social networking services such as Facebook, LinkedIn, bespoke SNS | Qualitative:16  Pilot: 8  Quantitative: 12  Mixed: 2 | No standardized outcome measures mentioned. Overviews only. | Overviews pointed toward a general improvement of loneliness after using social technology. | Increased use of internet led to moderate improvement in loneliness. | Systematic Review |
| Damant et al. (2016)[50] | Effect of technology use on the quality of life of older adults | 91 studies (10 databases, 2007–2014) | 4–9109 participants, aged 50+  Total Number of Participants Unclear | Mainstream ICT Remote care | No information supplied. | UCLA Scale | UCLA score down -2.84 after 3 months | Overall conclusion that internet use and videoconferencing reduces loneliness. | Scoping Review |
| Morris et al. (2014) [44] | Effect of smart technologies on social connectedness. | 18 studies (6 databases, 2000–2013) | 12–191 participants aged 66–82, Total no. of participants=2166 (1715 women and 451 men). | Computer/internet training and Internet-operated therapeutic software (Wii) | RCT: 12  Cohort: 6 | DJGLS and UCLAS | Among 18 studies, 17 were related to ICT and 1 was related to videoconferencing. 12 ICT studies reported that smart technologies improve loneliness and expand social networks. The videoconferencing study also yielded positive results. | Improvement in social support, loneliness, and social networks. Statistically significant improvement in loneliness found in 3 studies (UCLA scale and DJGLS). | Systematic Review and Appraisal of Literature |
| Husebø and Storm (2014)[55] | Virtual visits in community health provision and their impact on the health of older adults | 12 studies (3 databases, 2003–2012) | 8–130 participants aged 55+ along with healthcare providers  Total no. of participants: 227 | Videoconferencing to carryout healthcare | RCT:3  Pilot: 1  Quantitative: 2  Qualitative: 7 | The Loneliness  Questionnaire  (nine-item questionnaire, Geriatric Depression Scale) | Mainly overviews, along with 3 studies that clearly indicated that virtual visits increase social inclusion and reduce loneliness | Virtual visits through videoconferencing led to feeling of social inclusion and reduction in loneliness | Integrative Review |
| Cohen-Mansfield and Perach (2015)[24] | Looks at various interventions for reducing loneliness among the elderly | 34 studies (6 databases, 1996–2011) | 9–708 participants aged 55–93  Total Participant Count: >587 | Computer/internet training/use Videoconferencing Computer-generated exercise Robotics | RCT: 16 | 5 DJGLS, 1 Self-report, 12 UCLAS, 1 Interview Schedule, Arizona Social Support, Belief in External Control Scale, 2 LSNS. | 13 studies reported an increase in social access; 14 other studies showed improved social connectedness | 12 studies found that technology interventions effectively reduce  loneliness. A further 15 studies evaluated the interventions as  potentially effective. Technology-based interventions improved loneliness in both one-on-one and group settings | Systematic Review |
| Khosravi and Ghapanchi, (2016)[49] | Assessed the effectiveness of technologies developed to assist the elderly | 41 studies (4 databases, 2000–2015) | Number of participants unknown, aged 60+ | Computer/ internet training Robotics Computer-generated exercise | RCT: 8  Qualitative: 1 | QOL, Depression, Social Isolation Custom Assessment, UCLAS, Social Network Structure | 3 computer and internet use studies showed increased social access; 1 robotic study showed increase in social support | Online activities more effectively improved social isolation than robotics | Systematic Literature Review |
| Khosravi, Rezvani and Wiewiora, (2016)[47] | Review of various types of technologies and their effectiveness in alleviating social isolation among older adults | 35 studies (4 databases, 2000–2015) | 12–3075 participants aged 50–93  Total no. of participants: >6714 | Computer/Internet training Internet use SNS use Videoconferencing Robotics Computer-generated exercise Videogames | RCT: 8  Qualitative: 3 | 3 DJGLS, 1 Mastery Scale, 1 Self-report, 6 UCLAS, 1 Belief in External Control, 1 MSPSS (Multidimensional Scale of Perceived Social Support), 1 Perceived Control of Life, 2 Researcher Developed Scale, 1 Social Supportive, 1 Self Anchoring Scale, 1 Social Network Structure | Video games /PRISM were the most effective, followed by Tele-  Care, followed by General ICT followed by Robotics.  7 studies (6 computer and 1 internet training) showed increase in social access, 2 robotic studies and 1 gaming study showed increase in social support | Technological interventions had positive impacts on social isolation and loneliness. General ICT showed the most significant impact with 12/15 studies showing significant changes in levels of social isolation or loneliness. 6/7 studies on robotic interventions also showed reduction in loneliness. | Systematic Literature Review |
| Choi et al. (2012) [20] | Examined the effectiveness of computer and Internet training, interventions for reducing loneliness and depression in older adults. | 6 studies (10 databases, 2000–2012) | 12–60 participants aged 64–82  Total no. of participants: 390 | Computer/ internet training | RCT: 4  QE: 2 | 1 Geriatric  Depression Scale, Center for  Epidemiologic Studies  Depression Scale), 3 UCLA, 2 DJGLS | Reduction in Loneliness: Effect size=0.546 with a 95% CI of 0.033–1.059 (*Z*=2.085, *P*=.037) based on data from 353 older adults. | Meta-analysis showed a significant reduction in loneliness across studies. Effect size was 0.546 with a 95% CI of 0.033–1.059 (*Z*=2.085, *P*=.037) | Meta-Analysis Review |
| Poscia et al. (2018) [46] | Review of interventions for improving loneliness and social isolation in older adults | 20 studies (5 databases, 2012–2015) | Number of participants not known, all older adults | Computer/Internet training Robotics | RCT: 4  QE: 3  Pilot: 1  Mixed: 1  Qualitative: 5 | 3 DJGLS, 1 Italian version of Loneliness Scale, 2 UCLAS, 1 Friendship Scale, 2 LSNS (Lubben Social Network Scale), 1 Loneliness Literacy Scale, 1 Social Support List Interactions | 5 ICT related studies showed an improvement in social connectedness | The examined technological interventions significantly reduced loneliness in older adults | Updated Systematic Review |
| Cattan et al. (2005)[42] | Review of interventions for reducing and preventing loneliness and social isolation in older adults | 30 studies (12 databases, 1970–2002) | 93 participants, Mean age 71, Total no. of participants: 6,556 | Computer and Internet training/ use | RCT: 16  Non-RCT: 10 | DJGLS, UCLA, single-item loneliness customized questionnaire. | 16 RCTs and 10 non-RCT; 5/9 group interventions demonstrated significant reduction in loneliness. | Internet use is linked to reduced loneliness | Systematic Review |
| Gardiner et al. (2016)[41] | Review of interventions for improving loneliness and social isolation in older adults | 39 studies (6 databases, 2003–2016) | 12–85 participants aged 57–85, Total no. of participants unknown. | Computer/internet use/training Videoconferencing Robotics | RCT: 6  Survey: 2  Mixed: 1  Qualitative: 11 | 3 DJGLS, 1 Self-report scale, 7 UCLAS, 3 LSNS, 1 PCMR, 2 Researcher Created, 1 Social Network Activity, 1 Social Support Behavior. | 2 Computer and Internet training studies, 1 videoconferencing, 1 AAT and 1 Pet Robot study all showed improvement in social connectedness and support | Limited evidence that these interventions improve loneliness | Integrative Review |
| Bornemann (2014)[35] | Impact of ICT usage on social isolation and  loneliness in older adults. | 7 studies (14 Databases 2004–2014) | 12–57 participants aged >50 y, Total no. of participants:  455 | General ICT | RCT: 3  QE: 4 | DJGLS, UCLA-LS | Overall mean weighted effect size of loneliness from five studies was not  statistically significant for determining that ICT reduces loneliness in older adults (*Z=*1.60, *P* =  .11) even after controlling for heterogeneity (*Z*=0.44; *P*=.37). | ICT was ineffective for relieving loneliness. Authors recommend further research using latest technologies. | Systematic Literature Review |
| Stojanovic et al,(2017)[23] | Narrative review on the effects of ICT, Smart Technologies, and Computer Training on Social Isolation and Loneliness | 13 Studies (5 Databases 2000–2016) | Number of Participants not identified. Age >50 | Smart Technologies, Computer and Internet Training, General ICT | 13 Reviews | DJGLS, UCLAS, LSNS, Self-reported scale | 9 out of 13 studies found a positive impact of ICT on social connectedness. | ICT interventions helped participants to gain social support and engage in activities that reduced their loneliness. Smart Technologies and Computer and Internet Training had some impact on social connectedness. | Systematic Search and Narrative Review |
| Ibarra et al, (2020)[45] | Systematic Review on Technology-supported Interventions to  Improve Older Adult Social Wellbeing: Loneliness, Social Isolation,  and Connectedness | 25 Studies (1 Database 2000–2020) | 3–300 Participants aged 65–93. Total no. of Participant: 1776 | General ICT, Videoconferencing, SNS | 19 Quantitative Studies, including 5 RCTs;  6 Qualitative Studies | Hughes 3-items, UCLAS, Author-developed,  Questionnaire, MOS (Medical Outcome Study) DJGLS,  Cohen perceived  social support scale, Hawthorne  friendship scale,  LSNS, Social (egocentric)  network analysis  interviews,  social support  survey | All of the qualitative studies reported positive outcomes (decreased loneliness). Among the quantitative studies, 12 reported positive outcomes such as  decreased loneliness (n=9) and increased network size (n=3). 7 studies reported no significant differences. | ICT was found to be important in long-distance interactions. Off shelf interventions (Facebook, WhatsApp, Skype) were preferred over custom solutions.  Computers were the most popular interventions, closely followed by tablets. Usability, not formally an outcome, may have an impact on non-use, with uncertainty and fear playing a part; however, these obstacles can be overcome with training and use. | Systematic Literature Review |
| Olatz Gurrutxaga Lerma (2021)[57] | Systematic Review exploring different kinds of interventions to prevent loneliness of older adults living in nursing homes | 16 Studies (3 Databases 2010–2020) | 10 out of 16 studies focused on technological interventions. 9–100 participants aged >60. | mHealth, MIM, 3D virtual Reality, Robot Paro, Videoconferencing, Intercare home video calls. | 2 Mixed Method Studies, 2 Qualitative, 1 RCT, 1 Quantitative, 4 Quasiexperimental | UCLA, Geriatric Depression Scale, LSNS, Life Satisfaction Index, Barthel Index, Social Support Behavior Scale, MMSE, SF36, QoL-AD, CHQ-12 (Chinese Health Questionnaire), Meaning of Life Questionnaire, General Mattering Scale, GDS-15 | Significant improvements in 3D intervention group, positive reaction to robot, decreased loneliness when interacting with robot; decrease in loneliness at 1, 3, and 6 months with videoconferencing. Home care video calls helped socialization but the dropout rate was high and technology was confusing. | Videoconferencing, robots, and messaging apps effectively reduced loneliness in older adults in nursing homes. Group  interventions were found to be more effective | Systematic Literature Review |
| Williams et al. (2021)[43] | A rapid systematic review into interventions to reduce social isolation and  loneliness during COVID-19 physical  distancing measures | 58 Studies (6 Databases) | Only 11 studies dealt with technology interventions.  No. of participants: 40–580;  Age >60 y | 4 studies on Videoconferencing, 5 on Computer Training, 3 on Video Games | 7 RCT, 4 quantitative | UCLA, DJGL, HLS-3 (European Health Literacy Survey) | Videoconference studies found a significant reduction in loneliness and social isolation; Computer training found a non-significant result. Video gaming studies also found a significant positive impact on loneliness. | Good evidence that interventions targeting social isolation and loneliness were useful during shielding/social distancing measures. Educational and cognitive technological interventions were most effective, especially those that facilitated networking between peers. | Rapid Systematic Literature Review |
| Wiwatkunupakarn et al. (2021)[53] | SNS usage and its relationship to social  isolation, loneliness, and depression among older adults | 15 Studies (3 Databases) | 32–12,300 Participants;  Total no. of participants: 34,542;  Age >60 y | SNS | 5 RCT, 6 observational, 4 experimental | The Center for  Epidemiology Studies Depression Scale (CES-D) PHQ-2 (Patient Health Questionnaire), Beck’s Depression Inventory (BDI) The Mental Health Inventory-5 (MHI-5),UCLA | Limited evidence from experimental studies that SNS usage helps to reduce loneliness. Unclear evidence that SNS improve social isolation. To reduce social isolation, SNS may need to be supplemented with person-to-person communication. Technological training might also play an important role by improving the ability of older people to use social media. | Online interventions played a preventive role in social isolation, loneliness, and depression. During the pandemic social distancing measures, technology enabled users to maintain social connections, which may have ameliorated the effects of social isolation. Review found inconclusive results on SNS use and depression, social isolation, and loneliness. | Systematic Literature Review |
| Newman et al. (2021)[54] | Systematic Review into SNS and the experience of  older adult users | 21 Studies (6 Databases) | 41–2162 participants aged > 50 y;  Total no. of participants: 7509 | SNS | 6 Qualitative, 1 Mixed Methods, 11 Correlational/Cross-sectional, 3 experimental/cross-sectional | None described | Users of SNS felt more socially and intellectually engaged, particularly those using the functions on the site to a greater extent. This suggests that participants who were more active on SNSs reaped greater rewards for wellbeing.  Most papers were of low or medium quality, suggesting that the relationship between SNS use and wellbeing among older adults is currently inconclusive. | Results suggested that older adults mainly use SNSs to keep in touch with close family and friends rather than making new connections. Concerns about privacy were a common reason for non-use of SNS. Especially concerning were ownership of data and social privacy. | Systematic Literature Review |
| Choi and Lee (2021)[51] | Systematic Review of Effectiveness of ICT Interventions for reducing loneliness in Older Adults | 23 Studies (3 Databases) |  | Animal Robot, Interpersonal Communication, Mobile Robot, Exercise Game, Humanoid Agent,  Online Social Platform | 4 RCTs | UCLA, Social Support, DJGLS, Mental Wellbeing (WHO-5), Duke Social Support Scale, Life Satisfaction | 8 Studies focused on loneliness and social isolation. Loneliness was lower in the intervention group (3.33 ±2.16 to 1.00 ±1.26; *P*=.07) than in the passive group (3.57 ±6.10 to 0.8 ±2.77; *P*=.13). Loneliness was significantly lower among those in the intervention group (3.53 ±1.3 to 1.38 ±1.33; *P*<.001) than among those in the usual care group (3.59 ±1.23 to 4.00 ±1.32; *P*=.064), and there was a significant decrease in loneliness and social isolation (*P*<.01). | Several studies reported no statistically significant difference in loneliness between the intervention  and control groups. In general, ICT interventions improved loneliness, life satisfaction,  social support, quality of life, health outcomes, and other affective responses. They also effectively reduced social isolation and increasing social networking. Results proved that ICT interventions designed for older adults can effectively improve loneliness. | Systematic Literature Review |
