# Supplementary material for: Effectiveness of Technology Interventions in Addressing Social Isolation, Connectedness, and Loneliness in Older Adults: Systematic Umbrella Review
Source: JMIR Aging. 2022 Oct 24;5(4):e40125. doi: 10.2196/40125 (PMC9641519; doi:10.2196/40125)
Supplement: Multimedia Appendix 4 [file aging_v5i4e40125_app4.docx]

## **Appendix 4–GRADE Certainty of Evidence**

| **Category** | **Number of Reviews** | **Number of Primary Studies** | **Risk of Bias** | **Inconsistency** | **Indirectness** | **Imprecision** | **Other Considerations** | **Number of Participants** | **Effectiveness** | **Certainty**  **Assessment** |
| --- | --- | --- | --- | --- | --- | --- | --- | --- | --- | --- |
| Group vs One-to-One | 4 | 41 (29 Group Studies and 12 One-to-One) | Not serious | Not serious | Serious | Serious | Low number of studies, difficult to extract information from reviews, only one RCT found in PS | 445 | 27 Group Studies Effective, 7 One-to-One studies Effective | Very Low |
| Effectiveness of Technology Interventions | 17 | 242 | Not serious | Serious | Not Serious | Serious | Some reviews did not provide clear results, 85 RCTs, heterogeneity in results and outcome measures | >20,000 | 163 Effective 69 Not effective. | Moderate–Low |
| General ICT | 14 | 119 | Not Serious | Not Serious | Not Serious | Serious | Biggest category in reviews. 26 RCTs | >16316 | 86 Effective, 33 Not Effective | Moderate |
| SNS | 3 | 21 | Not Serious | Not Serious | Serious | Serious | Mixed results, lack of quantitative analysis, few RCT’s | >300 | 11 Effective, 10 Not Effective | Low |
| Videoconferencing | 8 | 14 | Not Serious | Not Serious | Not Serious | Serious | 6 RCTs | >500 | 7 Effective, 3 Non-effective | Moderate–Low |
| Mobile Instant Messaging | 1 | 3 | Not Serious | Not Serious | Serious | Not Serious | No RCTs, very few studies. | 114 | All Effective | Very Low |
| Computer and Internet Training | 12 | 66 | Not Serious | Serious | Not Serious | Serious | 51 RCTs, many unclear on whether it was the training element or technology use that caused the effect | >7000 | 37 Effective, 25 Not Effective | Low |
| Telecare | 4 | 34 | Not Serious | Serious | Serious | Serious | 4 RCTs, Small number of studies, heterogenous description of telehealth | >450 | 22 Effective, 12 Not Effective | Very Low |
| Robotics | 6 | 14 | Not Serious | Not Serious | Not Serious | Serious | 5 RCT, varied definition of robotics, heterogenous measures. | >200 | 11 Effective, 3 Not Effective | Moderate–Low |
| Gaming | 3 | 7 | Not Serious | Not Serious | Serious | Serious | No RCTs, 3 Studies, 1 Review | <50 | 1 Effective 1 Not Effective | Very Low |
| 3D and Augmented Reality | 1 | 1 | Not Serious | Not Serious | Not Serious | Not Serious | No RCTs, single study, few participants | <25 | 1 Effective | Very Low |
| Usability | 4 | 14 | Too little information | Too little information | Too little information | Too little information | No RCTs, not a formal measure, cursory mention | >150 | Too little information | Very Low |

**Key to GRADE Quality of Evidence Assessment**

| GRADE | Confidence |
| --- | --- |
| **High** | Further research is very unlikely to change our confidence in the findings |
| **Moderate** | Further research is likely to have an important impact on our confidence in the findings and may change the findings |
| **Moderate–Low** | Further research is likely to have an important impact on our confidence in the findings and is likely to impact the findings. |
| **Low** | Further research is very likely to have an important impact on our confidence in the findings and is very likely to change findings. |
| **Very Low** | Any estimate of effect is very uncertain. |
